# Antibiotic Resistance: Do We Need Only Cutting-Edge Methods, or Can New Visions Such as One Health Be More Useful for Learning from Nature?

**DOI:** 10.3390/antibiotics12121694

**Published:** 2023-12-03

**Authors:** Maria Vitale

**Affiliations:** Genetics of Microorganisms Laboratory, Molecular Biology Department, Istituto Zooprofilattico Sperimentale della Sicilia “A. Mirri”, 90129 Palermo, Italy; maria.vitale@izssicilia.it

**Keywords:** antibiotic resistance, biofilm and quorum sensing, antimicrobial peptides, monoclonal antibodies, phage therapy, bacterial predators, educational and socio-political aspects, sepsis, climate change, One Health perspective

## Abstract

Antibiotic resistance is an increasing global problem for public health, and focusing on biofilms has provided further insights into resistance evolution in bacteria. Resistance is innate in many bacterial species, and many antibiotics are derived from natural molecules of soil microorganisms. Is it possible that nature can help control AMR diffusion? In this review, an analysis of resistance mechanisms is summarized, and an excursus of the different approaches to challenging resistance spread based on natural processes is presented as “lessons from Nature”. On the “host side”, immunotherapy strategies for bacterial infections have a long history before antibiotics, but continuous new inputs through biotechnology advances are enlarging their applications, efficacy, and safety. Antimicrobial peptides and monoclonal antibodies are considered for controlling antibiotic resistance. Understanding the biology of natural predators is providing new, effective, and safe ways to combat resistant bacteria. As natural enemies, bacteriophages were used to treat severe infections before the discovery of antibiotics, marginalized during the antibiotic era, and revitalized upon the diffusion of multi-resistance. Finally, sociopolitical aspects such as education, global action, and climate change are also considered as important tools for tackling antibiotic resistance from the One Health perspective.

## 1. Introduction

The discovery of antibiotics was a milestone for the advancement of medicine throughout the world. Infections with pathogenic bacteria were causing severe diseases and several deadly epidemics before the diffusion of antibiotics. Furthermore, progress in modern medicine has been assured through the capacity to control infections in clinical settings. Without effective antimicrobials, modern medicine is at risk: minor and major surgeries, cancer chemotherapy, and transplants are all dependent on antimicrobials to prevent and treat infection in immunosuppressed patients. It has been estimated that up to 10 million people could die by 2050 due to the failure of antimicrobial treatments. Here, poorly treated bacterial infections can lead to failures of different organ systems, particularly in fragile patients, and excessive immune-mediated inflammatory responses during sepsis may occur more frequently https://www.sepsis.org/power-the-amrevolution/ (accessed on 3 November 2023). As in many other fields, the problems of AMR diffusion are often due to disequilibrium caused by human activity in the natural environment in which bacteria can immediately adapt due to their high genome plasticity. The prevalence and sometimes very rapid diffusion of MDR is largely due to the inappropriate use of drugs not only for clinical treatment in animals and humans but also for other purposes. Antibiotics were used in the past as growth promoters for livestock to increase food production and profit. Although this practice is now illegal in many countries, it is possible that this practice may be still be in place in some countries due to a lack of appropriate controls by public authorities. Antibiotic consumption is a key driver of antibiotic resistance. Antibiotics are often inappropriately prescribed for viral infections such as colds and influenza. The quantity and quality of antibiotic prescribing differs greatly among countries. For instance, a greater prevalence of resistant microorganisms exists in southern European countries where the amount of antibiotic consumption is about three times higher than in Scandinavian countries or the Netherlands [1]. After decades of antibiotic misuse, the result is that resistance is a huge problem for public health worldwide, as underlined by major international health organizations for humans and animals such as the WHO, FAO, and WOAH.

A multidisciplinary One Health approach has been advocated at a global level to tackle resistant bacteria, recognizing that human and animal health are interdependent and strictly related to the health of ecosystems. Resistant strains from humans and animals are dispersed in the environment, particularly when organic manure is used in agriculture or through sewage practices, so a continuous cycle is present from human activities to the environment. In this multidisciplinary approach, bacteria are analyzed from several sources (human, animal, and environmental), looking at the whole pool of resistance genes, “the resistome”, which includes all types of antibiotic resistance genes acquired via vertical transmission (intrinsic, taxa-specific) or those horizontally transferred, (taxa-nonspecific), as well as silent/cryptic resistance (phenotypically sensitive; genes present and functional but not expressed) and proto-resistance (phenotypically sensitive; little/no activity until mutated) [2]. In this review, an excursus of technical and scientific aspects is presented, ranging from antibiotic functions and resistance mechanisms to alternative ways to address antibiotic resistance, emphasizing “natural lessons”. Furthermore, from the perspective of “One Health” and a multidisciplinary approach, some socio-political aspects are briefly described in the belief that the only way to control antibiotic resistance and assure a high level of global health is to adopt simultaneous action and synergy, not only between different technical methods but also between different private and public stakeholders in civil society.

## 2. Antibiotics’ Primary Targets and Resistance Mechanisms

**Figure 1:**
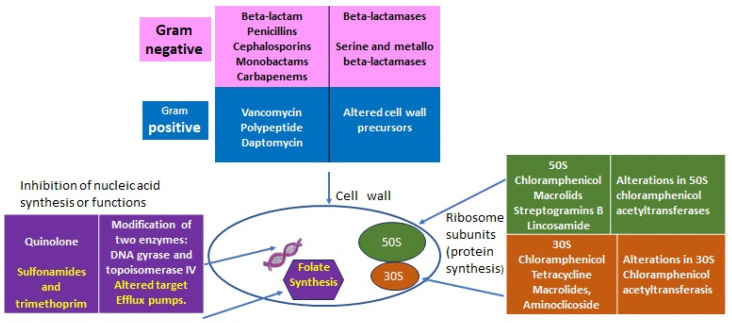
Classes of antibiotics with targets and some resistant determinants.

The discovery of penicillin in 1928 represented one of the great milestones in human medicine after centuries of periodically deadly epidemics due to bacterial infections amplified by wars, poverty, and poor hygienic conditions. Over time, antibiotics became very popular for their efficacy, specificity, and relatively low cost. Although some resistance was always possible, antibiotics were considered innocuous for humans and animals because their specific targets are present in bacteria and not in eucaryotes (Figure 1). In light of recent evidence of the great importance that the internal microbiota has for its respective host (animals, humans, and plants) this concept should be revisited, and a rational plan for the usage of antibiotics is important not only to control the resistance of pathogens but also to preserve the “good bacteria” to benefit the ecosystem and individuals’ health.

### 2.1. Lessons from Nature: From Intrinsic to Acquired Resistance

**Figure 2:**
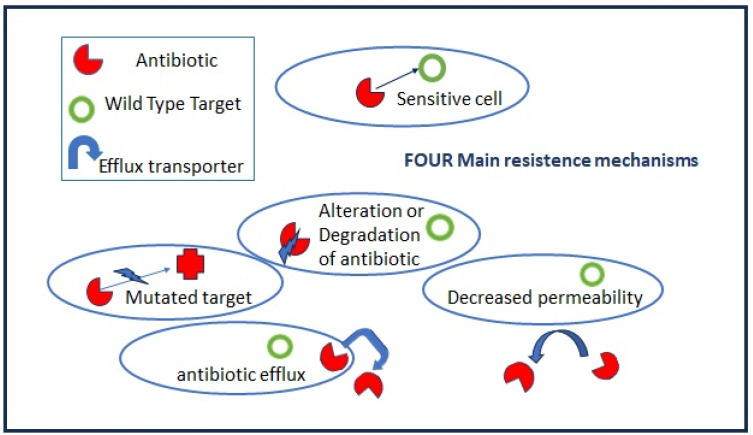
Antibiotic resistance mechanisms.

Antibiotic resistance is a natural and general phenomenon in many bacteria which carrying chromosomally located resistance genes (intrinsic resistance) to compete with other species in their environment and to be successful. Highly effective antibiotics have been derived from environmental microorganisms such as Streptomyces, Actinomycetes, and Fungi that produce antibiotics as secondary metabolites. The main molecular mechanisms of bacterial antibiotic resistance can be divided into (I) modifications to the target sites of antibiotics; (II) the alteration or degradation of the antibiotic; (III) antibiotic efflux via transporters; and (IV) reduced antibiotic penetration into bacteria through decreased membrane permeability (Figure 2).

#### 2.1.1. Intrinsic Resistance

All microorganisms are trained through evolution to become the fittest and best adapted to a particular environment, but they are also capable of innovating when alterations to their natural habitats occur [3]. Intrinsic resistance (IR) is an intraspecies genetic and phenotypic resistance which is transmitted vertically and has been used for decades to prepare selective culture media for specific enrichment and to isolate bacteria from complex matrices such as food, soil, and tissue samples. IR is present in the majority of the most relevant opportunistic pathogenic and resistant bacteria in clinical settings, including some bacteria of the so-called ESKAPE group (*Enterococcus faecium*, *Staphylococcus aureus*, *Klebsiella pneumoniae*, *Acinetobacter baumannii*, *Pseudomonas aeruginosa*, and *Enterobacter* species), due to their capacity to escape or evade the action of antimicrobial agents [4]. Moreover, these opportunistic pathogens also have great capacity to acquire new resistance through horizontal transfer and to form biofilms.

IR can exist for a single class of antibiotics, such as the resistance of *Listeria monocytogenes* to cephalosporins [5], but also for multiple drugs, such as the case of *Enterococci* (Gram-positive, monoderm), which have the potential for resistance to virtually all clinically useful antibiotics [6]. *P. aeruginosa,* among Gram-negative bacteria (diderm bacteria), represents well the phenomenon of intrinsic bacterial resistance since it carries practically all known mechanisms of antimicrobial resistance: the activation of chromosomal AmpC cephalosporinase; the production of plasmid- or integron-mediated b-lactamases from different molecular classes, diminished outer membrane permeability, and the overexpression of active efflux systems with wide substrate profiles, etc. [7] (Table 1).

#### 2.1.2. Acquired Resistance

Acquired resistance is related mainly to two mechanisms:

(a) Mutations in target genes located at chromosomal or extrachromosomal elements which are vertically transmitted in the same bacteria species. The mutation rate increases when bacteria are actively multiplying as, for example, during the acute phase of host infection.
(b) Horizontal gene transfers occur through mobile elements that can be transmitted both intraspecies and among different bacteria genera, i.e., the vancomycin-resistant gene (*vanA*) from *Enterococcus* to *S. aureus*. Plasmids, prophages, pathogenicity islands, restriction and modification systems, transposons, and insertion sequences are able to move within the host genome as well as jump across genomes. Mobile elements can change their insertion location and copy number and produce frequent gene gain and loss, modifying and co-evolving with chromosomal genomes. The genetic modifications induced by mobile elements can deeply affect bacterial fitness, contributing to their adaptation to new environments and, ultimately, producing evolutionarily distinct species over time. Once the acquisition of resistance determinants is established in few strains, antibiotic misuse and pressure drive the positive selection of resistant over sensitive strains.

The paradigm for this phenomenon is represented by the fast antibiotic resistance acquisition of *S. aureus* from ampicillin to methicillin to vancomycin in less than 60 years. In contrast to other bacteria, *S. aureus* was naturally susceptible to virtually every antibiotic that was ever developed, starting from penicillin. However, by the mid-1940s, only a few years after the introduction of penicillin into daily clinical practice, penicillin-resistant *S. aureus* strains were encountered in hospitals and, within a decade, had become a significant problem in the community [11]. Considering its ubiquity in the environment and its pathogenicity for humans and animals, as well as its antibiotic resistance and biofilm capacity, *S. aureus* is a major concern in both hospital and community settings.

#### 2.1.3. Acquired Resistance through Bacterial Cooperation

The study of biofilms as sources of persistent infection and contamination underpinned the importance of looking at microorganisms in natural conditions, as members of complex communities which modify their capacities. In contrast to laboratory conditions in which single colonies of the same strain are grown and analyzed in pure cultures, in natural external environments and internally in a host, bacterial cells are “compelled” to interact with many different components [12]. “Social structure” has been a fitness evolutionary advantage for “social animals” including humans passing from nomadism to settled communities. Individuals each gain a different “social role and activity”, and all work together for the wellness of the community. In bacteria, a cooperative resistance process has been observed when resistant bacteria producing beta-lactamases can help antibiotic-sensitive bacteria survive during antibiotic treatment [13]. Cooperative action has been observed in sensitive and resistant mixed cultures of *Salmonella tiphymurium* strains toward the antibiotic ceftriaxone with an increase in the MIC value upon an increase in cell density [14].

## 3. Lessons from Nature: Biofilms

Biofilms are formed when specific signals from bacteria concentrations and nutrient depletion are “communicated” among bacterial cells through “Quorum Sensing” (QS) molecules called Auto-Inducers (AIs) [15]. QS regulatory networks are very complex and can be divided into several categories, namely, AHL systems (N-acyl homoserine lactones) in Gram-negative bacteria and autoinducing peptides (AIPs) in Gram-positive bacteria [16]. In addition, two other systems, AI-2 and AI-3, have been found to be present in both Gram-positive and Gram-negative bacterial species and to participate in interspecies cross-talk. The first two systems have been extensively studied for many years, while AI-2 and AI-3 are still poorly explored [16,17].

The cell-density dependent regulation of gene expression defined as quorum sensing consists of at least four steps: (I) the synthesis of signal autoinducers (Ais), (II) the excretion of Ais, (III) at a certain threshold concentration of AIs, the activation of a specific receptor, and consequently, (IV) the activation or suppression of gene expression.

In the past two decades, QS inhibiting agents that can effectively inhibit biofilm formation in bacteria have been identified. The inhibitory mechanism can be divided into three categories based on the mode of action: (i) AI signaling molecules inhibit the production of autoinducers via the inactivation of signaling molecule synthases, the neutralization of AIPs with antibodies, and the modification or degradation of the signaling molecules in a quorum quenching (QQ) mechanism; (ii) targeting receptors inhibit the activity of AIs on regulated genes, and flavonoids and furanones can bind the receptors of many pathogenic bacteria; and (iii) targeting the downstream signaling cascade avoids all sequential steps such as the activation or suppression of gene expression for biofilm organization [18].

QS-inhibiting agents may be synthesized from derivatives of known Qs, via the modification of existing QQ enzymes, or from approved drugs that have QS-inhibiting activities [19].

Natural products have been investigated to identify potential new QS inhibitors. The natural plant-derived compounds trans-cinnamaldehyde (CA) and salicylic acid (SA) can significantly inhibit the expression of QS-regulated genes involved in virulence, rhamnolipids, and reduced biofilm formation in *P. aeruginosa* [20].

Marine bacteria are sources of many bioactive molecules and have also been explored in the search for molecules which can be active toward the pathogenicity and virulence of infectant bacteria in contrast to bactericidal activity [21]. Targeting QS causing virulence loss is beneficial for patient health, maintaining bacterial population balance and decreasing the positive selection of resistant bacteria upon the killing of sensitive bacteria.

In a biofilm, bacteria in different layers are differently active with respect to both metabolism and cell division so that antibiotics targeting active cells are ineffective on less active and quiescent cells (persister cells) which are able to reorganize the biofilm later on. Bacteria in a biofilm can become about 1000-fold more resistant compared to their planktonic state [22,23]. There are several mechanisms responsible for the higher resistance of bacteria in a biofilm:

(1) The extracellular polysaccharide matrix (EPS), which is produced upon biofilm organization, may slow down or impair antibiotic penetration.
(2) In biofilm microenvironments, metabolic byproducts, waste, and nutrients accumulate. Additionally, oxygen may be greatly reduced, creating an anaerobic environment. For example, low oxygen levels reduce the bactericidal effects of the antibiotics tobramycin and ciprofloxacin, while pH changes can negatively impact aminoglycoside’s action.
(3) Strict cellular contact and communication and the presence of a large amount of extracellular DNA in the biofilm EPS facilitate horizontal gene transfer from resistant to sensitive bacteria.
(4) The presence of different metabolic stages of bacteria in the community create an environment in which antibiotics that are active on dividing cells are ineffective toward more quiescent cells.
(5) The resistance of bacterial “Persister” cells: small subpopulations of bacteria that enter a “spore-like” state in which they are resistant to extreme conditions, like chemical treatment or antibiotic activity. These persisters exist in a dormant state without performing any genetic changes and do not divide in the presence of antibiotics. But once the organisms are released from the biofilm or begin dividing again, they return to their pre-persister susceptibility profile.

Biofilms represent a relevant crossroads for the One Health approach since they are involved in recurrent infections and persistent contaminations causing problems in animal and human health. Nosocomial infections related to medical devices contaminated with bacterial biofilms have been reported for many years in human medicine [24]. In contrast, the involvement of biofilms in animal health has been particularly notable in the last two decades. *S. aureus* biofilms have been observed in mastitis cases [25] and are involved in many other health problems in animals, such as wound healing in horses [26].

Regarding alternative ways to control pathogenic bacterial infections, we must also consider their capacity to be effective against biofilms by either preventing or destroying their formation. In addition, the possibility of the coexistence of different “weapons” in a synergistic action with conventional antibiotics can render infection treatment more efficacious in the near future. Synergic action can avoid an overuse of antibiotics, resulting in more effective low-dose therapy and decreasing the selection of resistant strains. As bacteria grow resistant to conventional antibiotics, alternatives should be deeply investigated, from antimicrobial peptides (AMPs) to monoclonal antibodies (MoABs) and from bacteriophages to predatory bacteria.

## 4. Lessons from Nature: The Power of the Host Defense—AMPs and MoABs

### 4.1. Host First-Line Defense: Antimicrobial Peptides

AMPs are an important part of the innate immune system (the first line of immune defense) present in all living organisms throughout the evolution from bacteria to humans, and their major mechanism relies on entering and destroying the cell membrane. AMPs with different structures form linear α-helical peptides, β-sheet peptides, linear extension structures, and both α-helix and β-sheet peptides [27]. AMPs with different activities from many organisms have been reported such as thionins, defensins from plants, and cathelicidins and defensins from mammals [28]. A continuously growing number of AMPs is reported in APD3 Antimicrobial Peptide Database https://aps.unmc.edu/ (accessed on 4 November 2023).

The activities of AMPs are mainly against bacteria but are also to a lesser extent against parasites, viruses, and fungi. Recently, a synthetic peptide from a freshwater red swamp crayfish showed good antifungal activity against *Candida albicans* with low toxicity for human cells [29]. Numerous studies have focused on AMPs from marine organisms, considering their rich biodiversity and the high level of facilitation of the diffusion of chemicals, antigens and biomolecules in marine environments that can highly stimulate innate immunity. Marine AMPs have been shown to be structurally different from their analogues in terrestrial species and often present novel structures [30]. AMP studies have been performed in organisms from the sea urchin and sea cucumber [31,32] to Mytilus [33].

Several AMPs are produced by bacteria, such as nisin and gramicidin from *Lactococcus lactis*, *Bacillus subtilis*, and *Bacillus brevis,* to control the growth of competitors. Bacterial AMPs are represented by bacteriocins and the recently discovered class of microcins.

Microcins, which are low-molecular-weight (<10 kDa) antibacterial peptides, are produced by different strains of Enterobacteriaceae to compete and best fit within the gut microbiota. So far, only 15 have been identified, displaying diversity in sequence, structure, target cell uptake and specificity, and cytotoxic mechanism of action. They are grouped into two classes: class I microcins are small (<5 kDa) and undergo extensive post-translational modifications; class II microcins are larger (~5–10 kDa) and are either unmodified except for disulfide bonds (Class IIa) or modified by the attachment of a C-terminal iron–siderophore which facilitates uptake (Class IIb). Growing evidence suggests that microcins may be adapted for therapeutic uses such as antimicrobial drugs, microbiome modulators, or facilitators of peptide uptake into cells [34].

#### Bacteriocins and Relative Resistance

Bacteriocins or AMPs from bacteria have been extensively studied for a long time in the food industry to preserve food from spoilage organisms. The best example is provided by the nisin produced by *Lactococcus lactis*. As seen for classical antibiotics, bacteria can develop resistance to AMPs, and both intrinsic and acquired resistance are present for bacteriocins. Resistance mechanisms involve (i) their reduction or loss of binding; (ii) sequestering; (iii) efflux pumping (export); and (iv) their degradation. A particular mechanism called “immune mimicry” has also been described as a bacteriocin-specific protection mechanism: non-bacteriocin-producing strains harbor “orphan immunity genes” by encoding functional homologues of bacteriocin immunity systems [35]. However, bacteriocin resistance is not very clearly defined, and its frequency cannot be predicted as it will depend on the specific strain and on growth conditions [36]. The high diversity and relative abundance of bacteriocins favor their use as alternative therapeutics in infectious disease management. A major advantage in the development of diverse applications is the fact that they are classified as GRAS (Generally Recognized as Safe) substances by the Food and Drug Administration (FDA) in the USA and by the European regulators of the pharmaceutical and food industries [37]. However, it is of paramount importance to address the issue of the emergence of resistance when bacteriocin-based antimicrobial strategies are proposed for clinical use. To date, knowledge of the development of bacteriocin resistance comes mainly from in vitro studies [38].

### 4.2. Antibodies Linked to Antibiotics

When examining the natural mechanisms at work during infection, it is well known that host–pathogen interactions are fundamental to disease evolution, which depends not only on a pathogen’s virulence but also on the host’s immune system defense. Following early innate immunity activation toward generic “invaders”, secondary defense mechanisms are triggered to increase the production of more specific antibodies against a particular pathogen. In addition, a pool of memory cells is saved as the “reserved arsenal” in case the same “invaders” reappear. This latter mechanism is exploited through the use of vaccines, which “mimic” natural infections to build a defense apparatus ready to prevent severe disease. As we definitively learned from the recent COVID-19 experience, vaccines are a critical tool in reducing the burden of infectious disease even when they do not completely block the contagion. However, vaccines will not be further discussed in this review.

The specificity of antibodies, and the inability of bacteria to develop resistance against them, make antibodies attractive, albeit expensive, alternative therapeutic agents. Natural antibodies have historically been used for therapeutic purposes by infusing the sera of recovered patients from different infections (serum therapy initiated by Kitasato and Behring more than 80 years ago). In the late 1980s, murine monoclonal antibodies were in clinical development thanks to the hybridoma procedures resulting from the fusion of B lymphocytes and myeloma cells. The first monoclonal antibody in clinical practice was a murine anti-CD3 monoclonal antibody, muromonab (OKT3), used for the treatment of organ transplant rejection [39]. However, murine monoclonal antibodies were often associated with allergic reactions and the induction of anti-drug antibodies. To overcome this problem, chimeric mouse–human antibodies were developed. MoAbs’ modes of action can be divided into anti-virulence, i.e., the neutralization of soluble toxins produced by pathogenic bacteria such as *Clostridiodes difficile* and *Bacillus anthracis,* and bactericidal, i.e., MoABs, against antigens of the bacterial membrane, such as the type III secretion system or against bacterial transporters resulting not only in neutralization but also in bactericidal effects [40]. Continuous biotechnology progress has resulted in the production of MoAbs for several diseases such as cancers, multiple sclerosis, and immune deficiencies [41,42].

## 5. Lesson from Nature: “Living Killers for Pathogens”—Phages and Predatory Bacteria

New approaches to overcoming AMR are represented by “living antibiotics”, such as bacteriophages or their components, and predatory bacteria.

### 5.1. Bacteriophages and Relative Resistance

Bacteriophages (phages), or viruses that kill or transform bacteria, were used as therapeutic approaches in the past, particularly in Eastern European countries, against severe infections such as dysentery before conventional antibiotics were discovered. Phages infect bacteria through their complementary receptor so that many such viruses are highly species-specific and have a low impact in disturbing normal flora. Upon binding to the receptors on the bacterial cell surface, phages inject their genetic material into the host cell and then either integrate this material into the bacterial genome (so-called “temperate” phages) or seize the bacterial replication machinery to multiply and lyse the cell (so-called “lytic” phages) [43]. In addition, they have the capacity to disturb bacteria organized in a biofilm through the presence of depolymerases on the tail structures of some phages that can degrade the extracellular matrix of biofilm-forming bacteria.

In the food industry, controlling contaminations by foodborne pathogenic bacteria is critical. Dangerous highly pathogenic strains can be found in livestock products, including enterohaemorrhagic *Escherichia coli* (EHEC; O157:H7), *Shigella* spp., *Enterococcus* spp., and *Listeria* spp. The multidrug-resistant pathogens most often isolated from human outbreaks, cattle, swine, and poultry are *S. aureus*, *Streptococcus* spp., *Vibrio* spp., and *Yersinia* spp. For food safety assurance, phage cocktails are used in food-processing systems, with several commercial preparations available for biocontrol against common foodborne pathogens such as *Salmonella* spp. SalmoFreshTM, and *Listeria monocytogenes* (ListShield™) [44]. The application of phage cocktails was also evaluated in fresh mixed-leaf salads in which Enterobacterales rods constituted a significant group of bacteria. Phage cocktails were applied through spraying or an absorption pad with a 100% reduction level of bacterial contaminants compared to a control sample after 48 h of incubation in the mixed-leaf salads, but a lack of effect in spinach samples may have been due to different bacterial species involved. A whole-spectrum phage cocktail application may constitute an alternative food microbiological quality improvement method without affecting food properties [45]. It is noteworthy that reducing contamination without the complete elimination of contaminant bacteria can be an efficacious method of assuring food safety regardless since bacterial load is important in disease evolution. However, a phage cocktail against a target pathogen may not be suitable in different niches such as animal-, plant-, or food-processing environments, so the “niche-specific” use of phages in the food processing system was recently proposed for the effective control of pathogens [46]. More field-scale studies are required to affirm the efficacy of phages in more real-world food production systems. In addition, bacteriophages can also be used for pathogen detection as biosensors in the food industry, i.e., the use of a bioluminescent reporter phage to detect *Bacillus anthracis* [47]. Recently, quantitative imaging of bacteriophage amplification for the rapid detection of *E.coli* in foods was proposed through T7 phage particles visualized using SYBR Green and a fluorescence microscope. The number of phage particles in the fluorescence images can be enumerated using image processing software, which allows for the rapid enumeration of phage amplification upon infection with the target bacteria. The phage quantification results showed a high correlation with the plaque assay method. The sensitivity of the imaging methods also eliminates the need for extensive sample preparation and the secondary amplification of the target phage’s DNA. This image-analysis-based rapid bacteria-detection approach can be further developed to be applicable for pathogen detection in agricultural industries and other foodborne pathogens [48].

In clinical practice, familiarity with phages is not well diffused in many countries. Although progress has been made, it is still considered only for atypical and severe infections such as multidrug-resistant strains in cystic fibrosis patients, severe wounds with prolonged hospitalizations, and pneumonia patients. Several issues need to be addressed, such as administration routes and appropriate dosage, before the use of a phage can be considered a first-choice treatment. It is important to select the appropriate phage with a high lytic capacity to kill bacteria but also to understand undesired toxic effects through the release of bacterial toxins in the target tissues/organs upon bacterial lysis that can stimulate the immune system. Although this negative aspect is shared with other protein-based pharmaceuticals such as live-attenuated vaccines, which are already used in human medicine [49], the application of bacteriophages in live animals or humans induces a cellular immune response which could lead to the inactivation of phages, rendering them ineffective in eliminating bacteria [50]. Some precautions need to be considered in massive phage preparations through the lytic cycle in infected bacteria. High-titer virus preparations contain also cell components, toxins, and DNA from the bacterial host, which must be removed before the phage can be injected to the patient. However, biotechnological advances have further expanded the repertoire of potential phage therapeutics to include novel strategies using bioengineered phages and purified phage lytic proteins [51]. Phages are very different from conventional antibiotics because of their capacity for “self-amplification”, which is a contributing factor in the efficacy of phage therapy; the process is self-limited once the hosts are no longer present. The real challenges for phage therapy are the fast-evolving resistance of bacteria and phage counter-defense mechanisms as parts the ubiquitous natural process of their co-evolution. Bacterial resistance can be due to mutations in phages’ receptors and the acquisition of CRISPR (Clustered Regularly Interspaced Short Palindromic Repeats) spacers, whereas counter measures by phages can rely on the modification of life cycle parameters (burst size, lysis time, etc.), mutating receptor binding proteins, or recombining with other viruses. This continuous struggle depends on several factors and timing such as the mutation rate, the genetic diversity of both populations, and the site and ecology of the infection, i.e., a mammalian gut versus other organs [52]. An important parameter for the phage–bacteria interaction is the fitness cost imposed by each defense and counter-defense mechanism. The adaptive immunity given by the CRISPR-Cas system as a resistance mechanism in *P. aeruginosa*, for example, is more costly for bacteria compared to receptor modification and, for this reason, will be selected and maintained only if the same phage often invades the same host. Thus, the presence of a phage defense mechanism (e.g., CRISPR-Cas) in a bacterium targeted for therapy does not always mean that it will be crucial for resistance to a therapeutic phage, especially if it is part of a diverse cocktail [53]. The use of phage cocktails to overcome a narrow host spectrum is also an advantage in lowering the possibilities of resistance evolution, but continuous monitoring during phage therapy in patients is necessary to control efficacy and to be assured that phage resistance is not developing [54]. In addition, several mechanisms are currently ongoing to reduce the chance of resistance development in phage therapy as described in a recent review [55].

Finally, an overview of clinical trials with phage therapy is present in a recent systematic review showing that the therapy is safe (with few and mild adverse effects) and highly efficacious, although there is heterogeneity in the reported studies [56]. With strong safety profiles, the main challenges of phage therapeutics involve strain variations among clinical isolates of many pathogens, battling phage resistance, and the potential limitations of host immune responses. However, the opportunities are considerable, with the potential to enhance current antibiotic efficacy, protect newly developed antibiotics, and provide a last-resort option in response to complete antibiotic failure [57].

### 5.2. Predatory Bacteria

A huge problem for antibiotic resistance is related to the growing and fast diffusion of beta-lactam resistance worldwide. For Gram-negative bacteria, one novel approach to treating infection is the use of living predatory bacteria such as *Micavibrio aeruginosavorus, Bdellovibrio bacteriovorus*, and other *Bdellovibrio* like organisms (BALOs) which are a phylogenetically heterogeneous group of small, rapidly swimming, Gram-negative procaryotic bacteria that are present ubiquitously in soil and aquatic environments. Predation is a natural and essential interaction present at all trophic levels and in all ecosystems, contributing to the maintenance of ecological balance [58]. Predation is usually associated with larger animals hunting and feeding upon smaller prey animals but, in the “microorganisms’ universe”, the smaller eats the larger. Although predatory bacteria have been researched by the academic community since the 1960s, only in recent years has the predation of *Bdellovibrio* versus Gram-negative pathogens been explored in biologically relevant systems to address important questions about host response, toxicity, and tissue damage. Their potential use versus resistant pathogens, in conjunction with the extremely fast diffusion of Gram-negative beta-lactamase-producing strains in the world, has triggered research toward a deeper understanding of their biology. In a range of animal models, the in vivo administration of *B. bacteriovorus* through different routes has demonstrated that they are safe for animals. In addition, as seen in phage treatment, they are also effective in bacterial biofilms [59]. Importantly, a number of multi-drug-resistant human clinical isolates have been shown to be susceptible to predation by *B. bacterivorous* [60]. Further work is needed to evaluate the dissemination of predatory bacteria from the administration site, along with the determination of any long-term effects of exposure for the host or their resident microbiota. Animal models are crucial but have limitations. Investigation of more established infections is needed, with support for defining a predatory bacteria dose range in relation to pathogen numbers, dose number, and correct schedule to treat the infection.

### 5.3. Main Comparisons of the “Two Living Antibiotic Classes”: Phages versus Predatory Bacteria

- Host/prey threshold: both phages and predatory bacteria cannot destroy the entire population of their host. The amplification process inside the host is self-limited, and it stops in the absence of the prey. However, this can also be an advantage for therapy since a reduction in pathogen load will reduce or eliminate disease symptomatology.
- Host range: Individual phages have high species-specificity ranges, and a rapid acquisition of bacterial resistance to phages often occurs in contrast to the broad bacterial prey range and the lack of simple resistance mechanisms.
- DNA transferance: Phages can transfer DNA through generalized and specialized transduction, with a potential risk for uncontrolled mutations which does not apply to predatory bacteria.

However, in contrast to phages that use bacterial machinery carrying only the genes for their own reproduction, the potential advantages of BALOs predatory bacteria are increased through the exploitation of the many useful enzymes and biomolecules they produce. Therefore, BALOs represent powerful platforms for biotechnology advancement in many fields [61].

## 6. Nature’s Lessons Need Receptive Students

The concept that the preservation of ecosystems is essential for human health and wellness is recognized in scientific communities but may not be common knowledge in general human society. Here, it is necessary to consider not only the technical–scientific aspects but also other societal aspects such as educational and socio-political aspects.

### 6.1. Education

Informative campaigns for a general audience, training, and antimicrobial stewardship practices are needed. To manage antibiotic resistance, continuous efforts to educate people about careful antibiotic use are important. Most educational efforts have been targeted to medical professionals to decrease antibiotic prescriptions and to properly conduct antibiotic therapy (correct dosage and length) through antibiotic stewardship programs which can sometimes be so heterogenous that results cannot be appropriately verified [62]. In addition, antibiotic stewardship programs for health professionals should point out the strict link between AMR and sepsis to improve the speed and accuracy of antimicrobial prescribing at an individual patient level. Improved confidence in the recognition of early sepsis, the faster determination of its etiology and antimicrobial susceptibility phenotype, and real-time surveillance through an AMR sepsis registry will lead to more effective coordination of clinical, laboratory, and public health AMR countermeasures [63,64].

Educational efforts have also been directed at adults, as the main consumers of antibiotics, sometimes without any medical suggestion. The use of antibiotics without prescriptions is unfortunately quite common in many countries in the world, ranging from less than 20% in Northern European and American countries to almost 100% in some African countries [65]. Moreover, it is difficult to change long-held views and behaviors regarding antibiotic use in adults. It may be a better strategy to reinforce educational programs in children and adolescents for two main reasons: (i) they have a naïve background and behaviors, and (ii) they are supervised by and in close contact with their parents and close relatives. This allows the relatives to receive the same educational messages, enlarging the network of knowledge about this important topic. Moreover, educational efforts to reduce antibiotic use must also include undergraduate students; when the knowledge, attitudes, and behaviors of medical professionals are ongoing, educating them about prudent antibiotic prescribing can be significantly effective in minimizing antibiotic resistance [66,67].

### 6.2. Social and Political

The global increase in multidrug-resistant infections and antibiotic failure in controlling pathogens has raised concerns in human and veterinary medicine. An official report of the European Food Safety Authority (EFSA) regarding zoonotic and indicator bacteria isolated from humans, animals, and food showed that a high proportion (28.6%) of human *Salmonella* strains were resistant to three or more antimicrobials, and 34.9% of E. coli strains isolated from pigs were resistant to more than six antibiotics [68]. Clearly, global action is necessary, involving specific authorities not only for appropriate regulation and control for public health but also to recognize how cultural and socioeconomic features, poor health literacy, low resources, and poor hygienic conditions may facilitate resistance spreading rapidly worldwide due to globalization. To control MDR, multi-state projects such as the EU-funded COMBINE project can be very important. This project aims to coordinate and support an international consortium managing AMR project delivery. It also provides communication across AMR projects through the establishment of an IT infrastructure to facilitate the collection, aggregation, storage, sharing, and analysis of preclinical and clinical data sets. Updated reporting is available at https://cordis.europa.eu/project/id/853967/reporting (accessed on 4 November 2023).

Further, national education ministries can help organize specific informative campaigns about MDR and the correct use of antibiotics at each level of education.

National policies need to be rapidly applied and reinforced worldwide to reduce pollution and global warming. Climate change also has a large impact on the diffusion of resistance in bacteria since bacterial evolution accelerates with global warming, and a higher production of bacteria leads to a higher chance of developing resistance. In addition, large population movements related to natural disasters (such as severe flooding or drought) or wars can lead to conditions of overcrowding and poor sanitation, which are also known to increase infection rates and genetic mutations and therefore antimicrobial resistance [69,70].

## 7. Future Directions

The bacterial universe is highly complex, and only deeper knowledge of its mechanisms can suggest the right tools to control antibiotic resistance. Alternative methods should not be limited to developing new classes of antibiotics derived from classical ones (i.e., multiple generations of cephalosporins) but should include exploiting the natural enemies or competitors of pathogenic bacteria. We have seen such practices in organic production in agriculture. Different alternatives to overcome antibiotic resistance will need to be as simple to use, effective, and low-cost as classical antibiotics. Continuous progress in biotechnology, bioinformatics, and “in silico studies” may solve the hindering aspects of these alternative methods. In addition, they can combine to provide synergistic approaches to help reduce the burden of resistance to classical antibiotics, preserving their effectiveness when clinically needed. On the host side, synergy among different disciplines, methods, and collaborative efforts at a multistate level are important for addressing the issue from a real One- Health perspective.

### Conclusions: The Beneficial Circle from Nature to Humans and Back to Nature

A deeper understanding of the interconnection and dependence of humans and other animals with a safe environment will hopefully lead to practical actions to reduce the negative impact of human activity in the planet. Knowledge of the biological elements necessary for understanding the evolution of AMR, including the microorganisms, host organisms (humans or animals), and environments involved in its emergence and dissemination, is an important step toward a comprehensive, multidisciplinary understanding of the problem in efforts to find a solution. Nature’s lessons suggest some solutions, but to increase their efficacy and safety, the continuous progress of biotechnology that only humans can facilitate is crucial. It is likely that multiple simultaneous approaches are needed for more practical methods to control resistant infections. As for many other health topics such as pandemics, pollution-related diseases, and the effects of global warming, humans need a new “Copernican revolution” at the global level to transition from an anthropocentric view to a “safe-ecosystem centric” view. Coordinated warnings from the international organizations for human (the World Health Organization, WHO) and animal health (the World Organization for Animal Health, WOAH), and food agriculture (the Food and Agriculture Organization, FAO) and environmental organizations such as the Global Alliance on Health and Pollution have already led to more broad international awareness. It is hoped that growing international and multidisciplinary collaborations under the “One-Health umbrella” will lead to global and effective actions for the future of the Earth.

## Figures and Tables

**Table 1:** Main determinants of intrinsic resistance in some pathogenic bacteria.

	Intrinsic Resistance (IR)	Determinants
All Gram-negative diderm bacteria	Glycopeptides, lipopeptides, and antibiotic targeting the bacteria peptidoglycan wall	EPS (extra-polymeric substance) avoids the permeability of antibiotics.
*P. aeruginosa*	Sulfonamides, ampicillin, 1st- and 2nd-generation cephalosporins, chloramphenicol, and tetracycline	Constitutive expression of Amp C beta-lactamase and efflux pumps. Low permeability of the outer membrane [7].
*Enterococcus* spp.	Aminoglycosides, cephalosporins, and lincosamides	Low cell wall permeability, aminoglycoside-modifying enzyme (AME), ribosome-modifying methyltransferase, altered cell wall, and ABC-efflux pump [6].
*L. monocytogenes*	Cephalosporins	Penicillin-binding proteins, multidrug resistance transporters, cell envelope proteins, etc. [4].
*E. coli*	Macrolides	Macrolides modifying genes such as mphA; efflux pump [8].
*K. pneumonia*	Ampicillin	SHV beta-lactamase, the fosfomycin resistance gene fosA, and the nalidixic acid efflux pump OqxAB [9].
*A. baumanii*	Cephalsporins, ampicillin, glycopeptides, and carbapenems	Class C (AmpC) and Class D beta-lactamases located in chromosome [10].
